# Effects of exogenous lactate administration on fat metabolism and glycogen synthesis factors in rats

**DOI:** 10.20463/pan.2020.0008

**Published:** 2020-06-30

**Authors:** Sunghwan Kyun, Choongsung Yoo, Takeshi Hashimoto, Hironori Tomi, Noboru Teramoto, Jisu Kim, Kiwon Lim

**Affiliations:** 1 Department of physical education, Konkuk University, Seoul Republic of Korea; 2 Department of Health and Kinesiology, Texas A&M University, Texas USA; 3 Faculty of Sport & Health Science, Ritsumeikan University, Shiga Japan; 4 Center for Regional Sustainability and Innovation, Kochi University, Kochi Japan; 5 Osaka Sangyo University Co., Ltd., Osaka Japan; 6 Physical Activity and Performance Institute (PAPI), Konkuk University, Seoul Republic of Korea; 7 Department of Sports Medicine and Science, Konkuk University, Seoul Republic of Korea

**Keywords:** Nutrition supplements, Lactate, Fat oxidation, Glycogen concentration, *FAT/CD36*, *PDH*, *CS*, *GYS2*

## Abstract

**[Purpose]:**

Lactate has several beneficial roles as an energy resource and in metabolism. However, studies on the effects of oral administration of lactate on fat metabolism and glycogen synthesis are limited. Therefore, the purpose of the present study was to investigate how oral administration of lactate affects fat metabolism and glycogen synthesis factors at specific times (0, 30, 60, 120 min) after intake.

**[Methods]:**

Male Sprague Dawley (SD) rats (*n* = 24) were divided into four groups as follows: the control group (0 min) was sacrificed immediately after oral lactate administration; the test groups were administered lactate (2 g/kg) and sacrificed after 30, 60, and 120 min. Skeletal muscle and liver mRNA expression of *GLUT4, FAT/CD36, PDH, CS, PC* and *GYS2* was assessed using reverse transcription-polymerase chain reaction.

**[Results]:**

*GLUT4* and *FAT/CD36* expression was significantly increased in skeletal muscle 120 min after lactate administration. *PDH* expression in skeletal muscle was altered at 30 and 120 min after lactate consumption, but was not significantly different compared to the control. *CS, PC* and *GYS2* expression in liver was increased 60 min after lactate administration.

**[Conclusion]:**

Our results indicate that exogenous lactate administration increases *GLUT4* and *FAT/CD36* expression in the muscle as well as glycogen synthase factors (*PC, GYS2*) in the liver after 60 min. Therefore, lactate supplementation may increase fat utilization as well as induce positive effects on glycogen synthesis in athletes.

## INTRODUCTION

Lactate is widely considered a waste product of anaerobic glycolysis (e.g. resistance training and high intensity exercise), and has been recognized to induce fatigue and muscular acidosis by lowering blood pH^1,2^. However, based on a recent study, lactate does not elicit dramatic acidosis or drastically alter body pH levels^3^. Moreover, lactate is not only a precursor molecule for gluconeogenesis but also an efficient source of energy through the Cori cycle and the enzyme lactate dehydrogenase (LDH)^4,5^. Rather, based on the lactate shuttle theory, lactate transporters such as monocarboxylate transporter families (MCTs) increase the intracellular transport of lactate, thereby contributing to reducing acidosis^6^. Indeed, MCTs transport lactate produced in glycolytic tissues through the blood stream to oxidative tissues which use lactate as energy source^7^. Therefore, lactate could be reconsidered as an efficient energy source in the body.

Recent studies have investigated, with a greater understanding, the energy-independent role of lactate. Interestingly, lactate administration affects metabolism-related factors. For example, Cerda-Kohler et al. reported that acute intraperitoneal injection of lactate (3 g/kg) affects the AMPK pathway, including the enzymes acetyl-CoA carboxylase (ACC) and TBC1 domain family member 4 (TBC1D4), which control oxidative metabolism factors in the mouse soleus muscle^8^. Moreover, phosphorylation of pyruvate dehydrogenase (PDH), which is regulated by 5' AMP-activated protein kinase (AMPK) and involved in the conversion of pyruvate to acetyl-CoA, was decreased after lactate injection into the mouse soleus muscle^8^. Furthermore, injection of 1 g/kg of sodium lactate increased blood lactate concentration by approximately 20 mM after 5 min, and increased the mRNA expression of peroxisome proliferator-activated receptor gamma coactivator 1-alpha (PGC-1α), a master regulator of mitochondrial biogenesis, in the mouse gastrocnemius muscle^9^. Lastly, Hashimoto et al. reported that long-term administration of a lactate-based supplement paired with exercise on mouse decreased scapular fat mass compared to the sedentary and exercise groups, causing a significant loss in body weight^10^.

Nevertheless, it is still unclear how lactate affects fat metabolism and glycogen synthesis over time. Therefore, the purpose of the present study was to investigate the effect of lactate administration on fat metabolism and glycogen synthesis factors in a time-specific manner.

## METHODS

### Animal care

Male 7-week-old Sprague Dawley (SD) rats were obtained from Raon Bio Inc. (Yongin, Korea) and cared for, following the ethical guidelines of the Animal Experiment Research Center of Konkuk University. Rats were fed ad libitum with a standard commercial diet and were housed in standard cages under controlled humidity (50 %) and temperature (23 ± 1 °C) with a 12 h light-dark cycle^11^. This study was approved by the Konkuk University Institutional Animal Care and Use Committee (No. KU18137).

### Study groups and treatments

**Figure 1.**
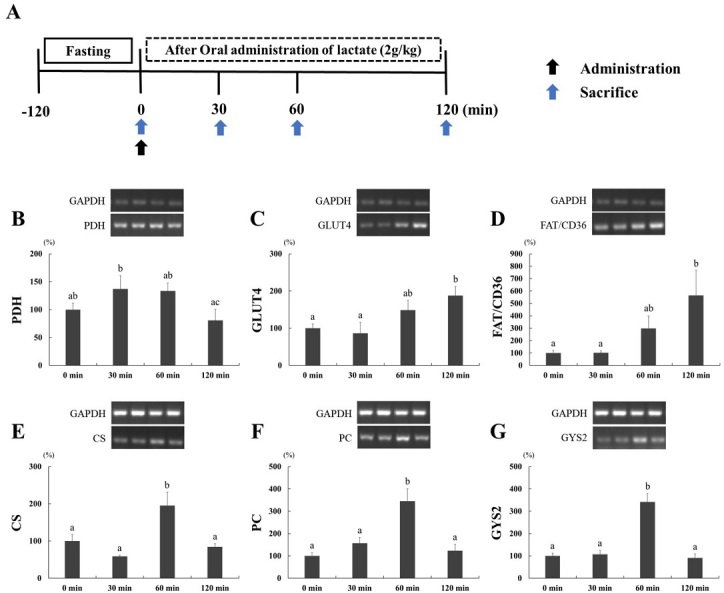
A. Experimental design. Rats were administered lactate after 2 hours fasting and sacrificed each time point. 0 min, 30 min, 60 min, and 120 min after lactate administration (2 g/kg) are referred to experimental groups. B-D. Effect of lactate administration on the mRNA expression in skeletal muscle. B. pyruvate dehydrogenase (PDH); C. glucose transporter type 4 (GLUT4); D. cluster of differentiation 36 (FAT/CD36). E-G. Effect of lactate administration on the mRNA expression in liver. E. citrate synthase (CS); F. pyruvate carboxylase (PC); G. glycogen synthase 2 (GYS2). Glyceraldehyde 3-phosphate dehydrogenase (GAPDH) was used as a mention for the normalization of the target mRNA expression. Significant differences between the groups are indicated alphabet and the values are presented the means ± SE (*n* = 6).

Rats were randomly assigned to one of four groups: 0 min (control, *n* = 6), 30 min (*n* = 6), 60 min (*n* = 6), and 120 min (*n* = 6). The group names indicate the time at sacrifice after lactate administration via an oral sonde. All rats were orally administered sodium lactate (2 g/kg) after fasting for 2 h. All groups were euthanized and dissected at each time point. Soleus muscle and liver tissues were obtained and stored at - 80 ℃ until further analysis (Figure 1A).

### mRNA expression analysis

mRNA expression was assessed by reverse transcriptase (RT) - PCR analysis. Total RNA from the soleus muscle and liver was extracted using QIAzol lysis reagent (QIAGEN, No.79306) and cDNA was synthesized using the amfiRivert cDNA Synthesis Platinum Master Mix (GenDEPOT, R5600). The RT reaction was performed using the following cycling protocol: annealing for 5 min at 25 °C, extension for 50 min at 42 °C, and RT inactivation for 15 min at 70 °C). To amplify the cDNA, Taq DNA polymerase (GenDEPOT, P0701) and specific primers were used. Cycling conditions were selected based on the primers (pre-denaturation for 2 min at 94 °C, followed by 25 – 40 cycles for 15 s at 94 °C, 30 s at 50 – 70 °C, and 1 min at 72 °C). The primer sequences used are listed in Table 1. mRNA expression was quantified using an ethidium bromide-stained 5% agarose gel.

**Table 1.** Primer sequences for RT-PCR.

Gene	Sequences
GAPDH	F–5’ TGC TGG TGC TGA GTA TGT CG 3’ R–5’ CTG AGT AGG CGC CAA TGA G 3’
PDH	F–5’ GCA GCA TTG TGG AAA TTA CCT 3’ R–5’ ACT TCG AAG GGT GGG TCA CT 3’
GLUT4	F–5’ CAG ATC GGC TCT GAA GAT GG 3’ R–5’ CTG AGT AGG CGC CAA TGA G 3’
FAT/CD36	F–5’ GCA ACA ACA AGG CCA GGT AT 3’ R–5’ AAG AGC TAG GCA TGG AA 3’
PC	F–5’ ACT TGT ATG AGC GGG ACT GC 3’ R–5’ TGA CCT TGA CGG GGA TTG GA 3’
CS	F–5’ CCG TGC TCA TGG ACT TGG GCC TT 3’ R–5’ CCC CTG GCC CAA CGT ACA TGC TC 3’
GYS2	F–5’ TTT CCT GGG AAG TGA CCA AC 3’ R–5’ TTT GCT GCA CAG AGA TAC CG 3’

Sequences for the identifying glyceraldehyde 3-phosphate dehydrogenase (GAPDH), pyruvate dehydrogenase (PDH), glucose transporter type 4 (GLUT4), cluster of differentiation 36 (FAT/CD36), pyruvate carboxylase (PC), citrate synthase (CS), and glycogen synthase 2 (GYS2) mRNA.

### Statistical analysis

IBM SPSS statistics 25 software was used to analyze data. Significant differences were determined using a oneway analysis of variance, followed by the least significant difference (LSD). Values of *p* < 0.05 were considered statistically significant and all results are presented as the mean ± the SE (*n* = 6).

## RESULTS

### Muscle mRNA expression

To test whether lactate administration affects metabolism factors, we examined mRNA expression in the soleus muscle. Lactate administration altered *PDH* expression, but this difference was not statistically significant compared to the 0 min control group (Figure 1B). The role of glucose transporter type 4 (*GLUT4*) is to regulate whole body glucose homeostasis. *GLUT4* expression at 120 min was significantly increased compared with 0 and 30 min (Figure 1C). The cluster of differentiation 36 gene (*FAT/CD36*), which regulates the uptake of fatty acid into cells, was increased at 120 min compared with 0 and 30 min (Figure 1D).

### Liver mRNA expression

We also examined mRNA expression in the liver to further elucidate the effect of lactate administration. We selected citrate synthase (*CS*) and pyruvate carboxylase (*PC*) for our analysis, which regulate metabolism by regulating the tricarboxylic acid cycle (TCA cycle). Figure 1E and 1F show that lactate administration significantly increased *PC* and *CS* expression at 60 min. The expression of glycogen synthase 2 (*GYS2*) was analyzed to investigate changes in glycogen synthesis in the liver. Interestingly, the expression of *GYS2* was significantly higher at 60 min compared to all other time points (Figure 1G).

## DISCUSSION

The purpose of the present study was to investigate the effect of exogenous lactate administration on fat metabolism and glycogen synthesis factors in a time-specific (0, 30, 60, 120 min) manner in the rat skeletal muscle and liver.

PDH is the major enzyme that converts pyruvate to acetyl-CoA, and thus increased expression of PDH indicates enhanced carbohydrate oxidation^12^. Cerda-Kohler et al.^8^ reported that treatment with lactate decreased *PDH* expression in the mouse soleus. However, our results did not show any significant differences in *PDH* expression at 30, 60 and 120 min after lactate administration in the rat soleus. This difference may be due to different methods of lactate administration. Additionally, we saw an increase in the expression of *GLUT4* and *FAT/CD36* 120 min after lactate administration. Glucose and fatty acids are the undisputable source of energy for the body. *GLUT4* and *FAT/CD36* are the major glucose and fatty acid transporters, respectively^13,14^, and it is interesting that acute administration of lactate significantly increased the expression of both genes. In a previous study, Jain et al.^15^ reported that muscle contraction significantly increased GLUT4 and FAT/CD36 protein levels. Moreover, Leick et al.^16^ reported that *GLUT4* and *FAT/CD36* were induced in a phase of recovery after endurance exercise in human skeletal muscle. Furthermore, a previous study showed that exercise training increased *GLUT4* and *FAT/CD36* while also enhancing the rate of fat oxidation^17-20^. Therefore, we assumed that oral administration of lactate induced an increase in fat oxidation rate in skeletal muscle.

To explore the effect of lactate administration on the liver, we analyzed select mRNA (*CS, PC* and *GYS2*) expression. In the first reaction of the TCA cycle, CS converts acetyl-CoA and oxaloacetate into citrate^21^. Siu et al.^22^ reported that CS is often used as a marker of metabolic rate regarding oxidative and respiratory capacity. Furthermore, PC converts pyruvate into oxaloacetate, which fuels the TCA cycle with its intermediates^23^. Accordingly, our results showed that lactate administration significantly increased *CS* and *PC* expression at 60 min, which could imply that lactate treatment enhances oxidative capacity.

In addition, the present study showed that lactate administration significantly increased *GYS2* at 60 min compared to 0, 30, 120 min. GYS2 converts glucose into glycogen and is the key enzyme for glycogenesis in the liver^24^. Wilamowitz-Moellendorff et al.^25^ reported that GYS- knockout mice have a significantly decreased glycogen concentration in liver. Pursell et al.^26^ showed that inhibition of *GYS2* expression restrained glycogen accumulation in the mouse liver. Therefore, lactate administration may enhance glycogen synthesis in the liver.

A limitation of this study is that we did not treat the rats with other doses of lactate or confirmed the effectiveness of an acute treatment. In addition, only mRNA levels and blood analysis were performed. Therefore, in future studies, glycogen concentrations in the muscle and liver should be assessed and the expression of more markers of glucose and fat metabolism should be quantified.

Nevertheless, we confirmed an increased expression of metabolic and glycogen synthesis enzymes such as *CS* and *PC* at 60 min after lactate administration. Moreover, a single bout of lactate administration enhanced the expression of *GLUT4* and *FAT/CD36* at 120 min after treatment. Lastly, the expression of *GYS2* was increased, which could result in glycogen accumulation in the liver. In conclusion, we suggest that lactate supplementation may increase fat utilization as well as have positive effects of glycogen synthesis. Further studies are needed to clarify the long-term effects of lactate on exercise capacity in athletes.

